# Isolation, Purification and Characterization of Vinblastine and Vincristine from Endophytic Fungus *Fusarium oxysporum* Isolated from *Catharanthus roseus*

**DOI:** 10.1371/journal.pone.0071805

**Published:** 2013-09-16

**Authors:** Ashutosh Kumar, Deepak Patil, Pattuparambil Ramanpillai Rajamohanan, Absar Ahmad

**Affiliations:** 1 Division of Biochemical Sciences, CSIR-National Chemical Laboratory, Pune, India; 2 Central NMR Facility, CSIR-National Chemical Laboratory, Pune, India; RMIT University, Australia

## Abstract

Endophytic fungi reside in a symbiotic fashion inside their host plants, mimic their chemistry and interestingly, produce the same natural products as their hosts and are thus being screened for the production of valuable compounds like taxol, camptothecin, podophyllotoxin, etc. Vinblastine and vincristine are excellent anti-cancer drugs but their current production using plants is non-abundant and expensive. In order to make these drugs readily available to the patients at affordable prices, we isolated the endophytic fungi from *Catharanthus roseus* plant and found a fungus AA-CRL-6 which produces vinblastine and vincristine in appreciable amounts. These drugs were purified by TLC and HPLC and characterized using UV-Vis spectroscopy, ESI-MS, MS/MS and ^1^H NMR. One liter of culture filtrate yielded 76 µg and 67 µg of vinblastine and vincristine respectively. This endophytic fungal strain was identified as *Fusarium oxysporum* based upon its cultural and morphological characteristics and internal transcribed spacer (ITS) sequence analysis.

## Introduction

Endophytic fungi are symbiotically associated with plants and can synthesize the same bioactive compounds and natural products as their host plant themselves, suggesting the possibility of intergeneric genetic exchange between the plant and the fungus; meanwhile causing no damage to the host. One of the best examples is that of the discovery of plant hormone gibberellins from *Gibberella fujikuroi* in the early 1930's wherein the pathways of gibberellin biosynthesis in the fungus and higher plants were found to be identical upto GA12 [1]. These findings ignited the prospect that the endophytic fungi associated with *Taxus brevifolia* plant too may be able to produce taxol [2]. Plant based drugs from a microbial source like endophytic fungi will be of immediate interest to pharmaceutical industries as these will help in getting rid of the several geographical and political barriers associated with transportation of plants as well as from the various environmental conditions which can hamper the quality and production of desired compounds. Microbial fermentation has several advantages over using parts of the plants for the production of drugs and bioactive substances as this can easily be carried out in tank fermenters, providing unlimited supply of drugs and negating the requirement of plant parts. Moreover, different stronger derivatives of the drugs can be obtained by altering the culture conditions. Also, the microbial extraction procedures are very easy and require less solvent in order to purify the drugs.

In the present era of global warming and the ecosystem hanging from a loose thread, this discovery comes as a boon as all the drugs for which plants were earlier being exploited can now be synthesized from the endophytic fungi associated with the plants itself, thus reducing the requirement of any other part of the plant and sparing them from extinction in most places. This proves to be true for a very wide extent of plant associated endophytic fungi and has opened up a new area for research and development wherein the drugs could be made available to all at low costs and help in recovering the balance of nature too.

Eversince, a number of endophytic fungi have been isolated from other *Taxus* plants such as *Taxus chinensis* [3], *Taxus wallichiana* [4] and *Taxus cuspidate* [5] which also produce Taxol and related compounds. Our group also has isolated endophytic fungi from Indian yew tree *Taxus baccata* and successfully screened them for Taxol production [6]. Another anticancer drug Camptothecin which was initially isolated from the wood of *Camptotheca acuminata* [7] can now be isolated from the endophytic fungus *Fusarium solani* which is associated with the plant [8]. Similarly, Podophyllotoxin, a well known anti-cancer drug is now being isolated from the endophytic fungus *Phialocephala fortinii* instead of its host plant *Podophyllum* (*Sinopodophyllum*) *peltatum* which was used in the past [9].

Vinblastine and vincristine, the wonder drugs for cancer, are being isolated from the leaves of field grown *Catharanthus roseus* plant by techniques such as tissue culture [10], cell culture [11], shoot culture [12], semi synthesis [13] as well as total synthesis [14]. Although the drugs can be obtained by these methods, their supply is limited and cannot meet the present requirements. A number of endophytic fungi have been isolated by Kharwar *et al.* from the *Catharanthus roseus* plant found in India [15]. But, there are so far no reports of vinblastine and vincristine from above endophytic fungi. Zahng *et al.* [16] and Tung *et al.* [17] reported that vincristine is produced by *Fusarium oxysporum*, an endophyte of *Catharanthus roseus* while Guo and Kunming [18] obtained vinblastine from *Alternaria* sp. isolated from the same plant found in China and showed the production based upon TLC and HPLC only.

In order to provide these novel drugs i.e. vinblastine and vincristine in abundance and at reasonable rates to the patient, and at the same time considering our responsibilities towards the conservation of nature, we decided upon the isolation, purification and characterization of these drugs from the endophytic fungus *Fusarium oxysporum* isolated from *Catharanthus roseus* found in India. The purification processes were performed with preparative TLC and HPLC and the characterization was done by UV-Vis, ESI-MS and ^1^H NMR spectroscopy.

## Materials and Methods

### Isolation, purification and maintenance of the endophytic fungus producing vinblastine and vincristine

Endophytic fungi were isolated from the leaves of *Catharanthus roseus* obtained from different areas of Pune (CSIR-National Chemical Laboratory and University of Pune, India); no specific permissions were required for these locations and the field studies did not involve endangered or protected species. The isolated endophytic fungus, *Fusarium oxysporum* maintained on potato dextrose agar (PDA) slants have optimum growth at pH 7.0 and temperature 27°C. Subculturing was done at monthly intervals to maintain the stock cultures and preserved at 15°C. Starting materials for fermentation experiments were taken from an actively growing stock culture, which were subcultured on fresh slants and incubated for 7 days at pH 7.0 and temperature 27°C.

### Identification of the endophytic fungus producing vinblastine and vincristine by cultural, morphological and molecular methods

Identity of endophytic fungal culture producing vinblastine and vincristine was established by using cultural, morphological and molecular approaches. For studying the cultural and morphological characters, the fungus was grown on PDA medium. Cultural characters such as color and nature of the growth of the colony were determined by visual observation. Morphological characteristics of the fungus like mycelia, conidiophores and conidia were microscopically studied (Carl Zeiss Axiovert 25 inverted microscope). Mycelia, conidiophores and conidia produced by the fungus in the culture was examined under a microscope. The genomic DNA of the endophytic fungal strain was extracted according to the method described by Lodhi *et al.* (1994) with slight modifications. ITS regions of genomic DNA of the fungus was amplified using ITS1- TCCGTAGGTGAACCTGCGG (forward primer) and ITS4- TCCTCCGCTTATTGATATGC (reverse primer). The purified PCR products obtained using ITS1 and ITS4 were sequenced using Sanger's dideoxynucleotide chain termination method and was carried out at Chromous Biotech Pvt. Ltd. Bangalore, India. The sequence obtained was further analyzed for its homology by using the online tool nucleotide BLAST.

### Isolation, purification and characterization of vinblastine and vincristine from the endophytic fungus *Fusarium oxysporum*

A two stage fermentation procedure was employed for the isolation of vinblastine and vincristine by *Fusarium oxysporum*. In the first stage, 500 ml Erlenmeyer flasks containing 100 ml medium (MGYP, (0.3%) malt extract, (1.0%) glucose, (0.3%) yeast extract and (0.5%) peptone) were inoculated with 7 days old culture and incubated at 28°C on a rotary shaker (240 rpm) for 4–5 days, which was used as seed culture (I stage). Later, 10 ml seed culture was transferred to 500 ml Erlenmeyer flask containing 100 ml production medium called as vinca medium-1 (Glucose: 3%, Succinic acid: 1%, Sodium benzoate: 100 mg, Peptone: 1%, Magnesium sulphate: 3.6 mg, Biotin: 1 mg, Thiamine: 1 mg, Pyridoxal: 1 mg, Calcium pentothenate: 1 mg, Phosphate buffer: 1 ml (pH 6.8), L-Tryptophan: 0.1%, Geranium oil: 0.05%.) which were incubated at 28°C for 20 days as shake culture (II stage), after which it was harvested and used for further study. Culture filtrates and mycelia were separated with the help of muslin cloth and then lyophilized. Lyophilized culture filtrate was extracted using ethyl acetate as a solvent system. The organic layer was separated from the aqueous layer using separating funnel. The extraction was repeated thrice and the solvent was dried using anhydrous sodium sulphate and concentrated under vacuum using rotavapour at 40°C in order to get crude extract. A small amount of crude extract was dissolved in ethyl acetate and subjected to thin layer chromatography (TLC) on silica gel-G (0.5 mm thickness) using chloroform∶methanol (8∶2) as a solvent system. The TLC plates were sprayed with ceric ammonium sulphate reagent. Vinca alkaloids spots produced brilliant violet color as well as purple color with above spraying reagent. Purification of fungal vinblastine and vincristine were done by silica gel column chromatography. The crude extract was loaded on silica gel column (60–120 mesh size, 40 cm×2 cm length width) pre-equilibrated with chloroform and eluted with a gradient of chloroform∶methanol (100% chloroform, 9∶1, 8∶2, 7∶3, 1∶1 and 3∶7 and 100% methanol). Fractions containing compounds with Rf values similar to that of the standard vinblastine and vincristine were pooled and subjected to preparative TLC on a 0.5 mm thick (20 cm×20 cm) silica plate and developed in chloroform∶methanol (8∶2) solvent system. The putative bands of fungal vinblastine and vincristine were scraped and eluted out with methanol. Purity of the isolated compounds was checked on TLC in the solvent systems such as (a) chloroform∶methanol (8∶2) (b) chloroform∶methanol (9∶1) and (c) ethyl acetate: acetonitrile (8∶2).

### Purification and quantification of vinblastine and vincristine by HPLC

Purity of fungal vinblastine and vincristine was determined by HPLC using C18 symmetry column (Waters). Sample (40 µg) was taken in 40 µl acetonitrile, injected in HPLC column and gradient elution was performed using 5%–95% acetonitrile in water with 0.01% trifluoroacetic acid at flow rate of 0.5 ml/min. A dual wavelength recorder set at 220 nm and 254 nm was used to detect the compound eluting from column. The absorption maximum of the purified compound was determined by Shimadzu PC 101 spectrophotometer. Sample was dissolved in HPLC grade methanol and spectral data was collected over 200–700 nm range. A 2 mg/ml concentration of a standard vinblastine and vincristine solution were prepared in HPLC grade acetonitrile. 50 µl of the above standard solution (2 mg/ml) plus 950 µl of HPLC grade acetonitrile was taken to make the final concentration of 100 µg/ml. 10 µl of this solution was injected into HPLC and analysed. Similarly, in order to quantify vinblastine and vincristine present in 1 litre of the culture filtrate, the culture filtrate obtained after 20 days was extracted and purified by HPLC. The purified fungal vinblastine and vincristine obtained were dissolved in 1 ml of HPLC grade acetonitrile and then 10 µl each of these purified solutions was injected into HPLC and analysed. The data of the area peak vs. concentration of the standards obtained were used to estimate the quantity of fungal vinblastine and vincristine in one liter culture filtrate.

### Electrospray ionization mass spectrometry (ESI-MS) and Tandem mass spectrometry (MS-MS) analysis

Molecular mass of the purified compounds was determined by M/S applied Biosystems APIQSTAR pulsar (ESI-MS) mass spectrophotometer. Samples for the analysis were dissolved in HPLC grade methanol, water, acetic acid in the ratio of 50∶50∶0.1. Samples were then analyzed by infusion method (injected into MS) at flow rate of 5 µl/min and at an IS voltage of 3800 V in TOF mode. Spectrum from a range of m/z 100–1400 Dalton was obtained. Fragmentation of the desired molecule was obtained by acquiring the product ion spectrum using MS-MS with similar parameters as used for ESI-MS. Spectrum from a range of m/z 100–900 Dalton was obtained. Molecular ions of the standard vinblastine and vincristine were also obtained for comparison.

### Nuclear Magnetic Resonance (^1^H NMR) analysis

^1^H NMR analysis of vinblastine was carried out on a Bruker AV 400 FT spectrometer operating at 400 MHz for ^1^H. Sample was dissolved in CDCl_3_ and measured with a spectral width of ∼8200 Hz, acquisition time of 1.82 s and a relaxation delay of 1.0 s was applied using a 30 degree flip angle. 256 and 124 transients were collected for A and B, respectively. Similarly, the ^1^H NMR analysis of vincristine was carried out on a Bruker AV 400 FT spectrometer operating at 500 MHz for ^1^H. Sample was then dissolved in CD_3_OD and measured with a spectral width of ∼8200 Hz, acquisition time of 1.82 s and a relaxation delay of 1.0 s was applied using a 30 degree flip angle. 256 and 256 transients were collected for A and B, respectively.

## Results

### Isolation, purification and identification of endophytic fungus producing vinblastine and vincristine

**Figure 1:**
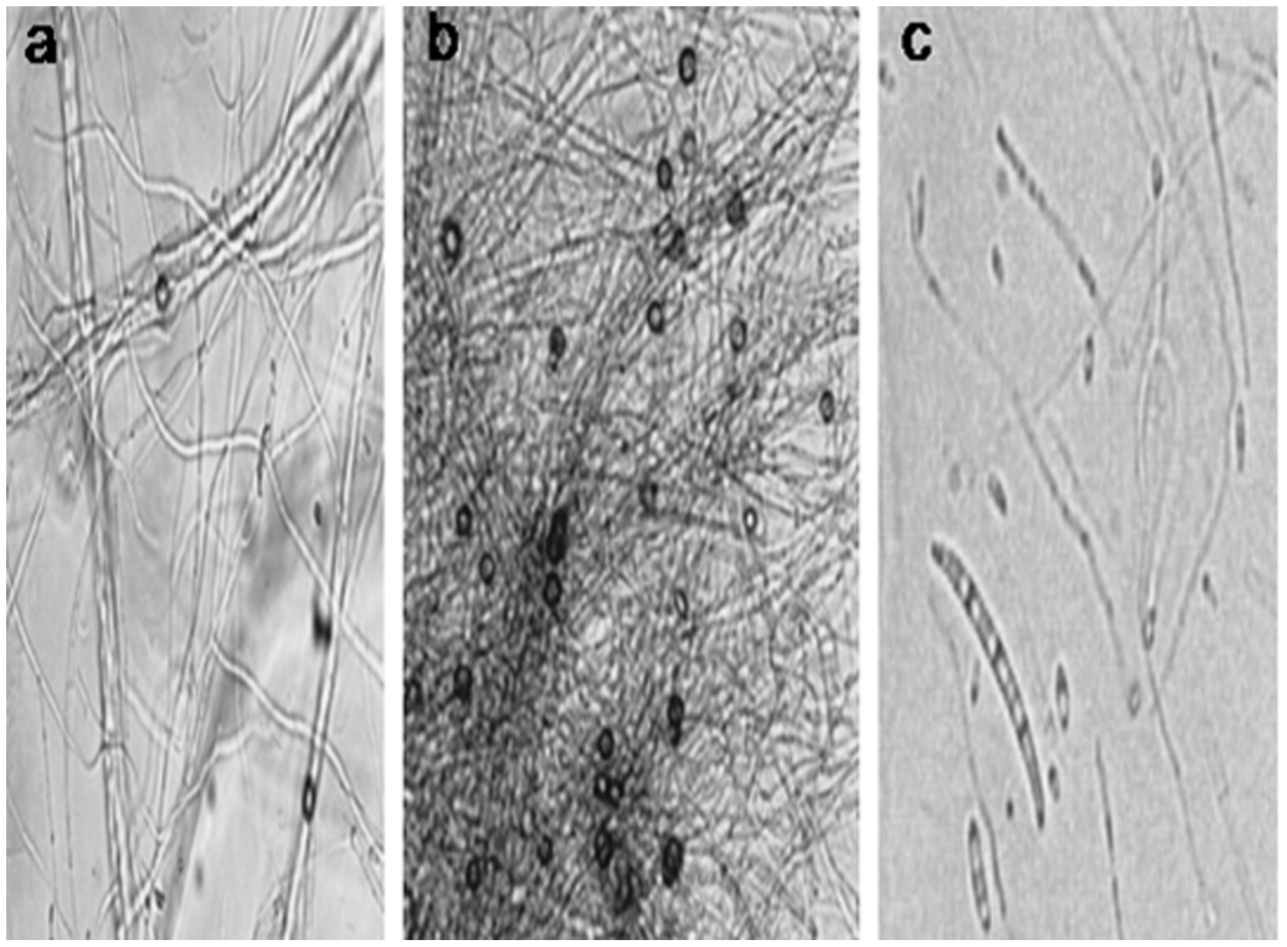
Morphological features of endophytic fungus *Fusarium oxysporum.* Colony shape: (a) Mycelium, (b) Chlamydospores (c) Macroconidia, microconidia and chlamydospores formed on PDA.

The fungal strain AA-CRL-6 which produces vinblastine and vincristine was identified using cultural, morphological and molecular approaches. The fungus AA-CRL-6 grown on PDA medium produced slow growing snow-white colonies. The strain also produced three types of spores called macroconidia, microconidia and chlamydospores (Figure 1) [19]. Most of the morphological characters of the strain (AA-CRL-6) agree with the known features of *Fusarium oxysporum*. Amplification of fungal genomic DNA by primers ITS-1 and ITS-4 yielded 487 bps fragments. The analysis of sequence revealed 99.9% identity with that of *Fusarium oxysporum*. The cultural, morphological and molecular studies confirm that the isolate AA-CRL-6 belongs to genus *Fusarium* and species *oxysporum*.

### Vinblastine and vincristine from culture filtrates

**Figure 2:**
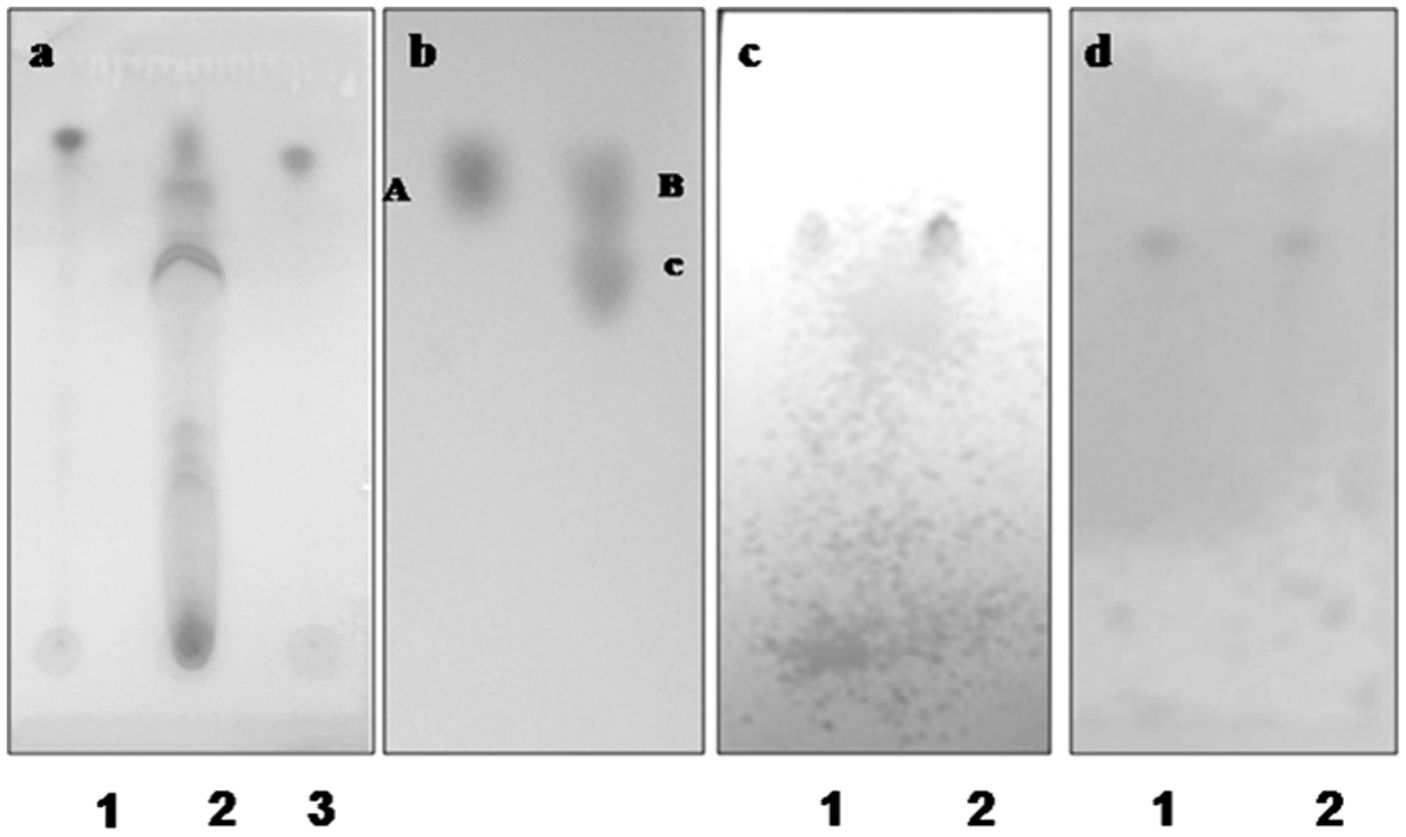
TLC analysis. (a) TLC of crude fungal vinblastine from culture filtrates along with standard Vinblastine 1: Vinblastine standard, 2: Crude sample, 3: Vincristine standard, Detection: Ceric ammonium sulphate reagent. (b) TLC of partially purified fungal vinblastine from culture filtrates along with standard vinblastine on silica gel using chloroform∶methanol (8∶2) solvent system. A: Standard vinblastine B: Partially purified vinblastine C: Partially purified vincristine, Detection: Ceric ammonium sulphate reagent. (c) TLC of fungal vinblastine purified from culture filtrates along with standard vinblastine on silica gel using chloroform∶methanol (8∶2) solvent system. 1: Purified fungal vinblastine 2: Standard vinblastine, Detection: Ceric ammonium sulphate reagent. (d) TLC of fungal vincristine purified from culture filtrates along with standard vincristine on silica gel using chloroform∶methanol (8∶2) solvent system. 1: Standard vincristine, 2: Purified fungal vincristine, Detection: Ceric ammonium sulphate reagent.

The culture filtrates, when extracted with ethyl acetate yielded a brown residue after the removal of solvent [20]. The substance was named as crude extract. The crude extract, upon TLC using silica gel G and chloroform∶methanol (8∶2) solvent system, produced five clearly differentiated brilliant purple colored spots when sprayed with ceric ammonium sulphate reagent (Figure 2a) [21]. The Rf values of the spots (0.77 for vinblastine and 0.74 for vincristine) derived upon TLC of the crude extract using chloroform∶methanol (8∶2) solvent system when compared with the standard Rf value of vinblastine and vincristine were found to be identical. The spots of vinblastine and vincristine in the crude extract showed identical color as with the standard when sprayed with ceric ammonium sulphate reagent. In addition to vinblastine and vincristine, three vinca alkaloids present in the crude extract as evident from TLC could not be identified. For the purification of vinblastine and vincristine from culture filtrate, the crude extract were fractionated on silica gel column with chloroform∶methanol (100% chloroform, 9∶1, 8∶2, 7∶3, 1∶1 and 3∶7, and 100% methanol). Partially purified vinblastine and vincristine with chromatographic properties similar to that of the standard vinblastine and vincristine (Figure 2b) were obtained. The partially purified vinblastine and vincristine were subjected to preparative TLC which yielded considerably pure compounds. The vinblastine obtained from preparative TLC showed single dark purple spot that later turned to very dark purple (within Rf value 0.77, Figure 2c) and the vincristine obtained from the same method showed single dark violet spot (within Rf value 0.74) when sprayed with ceric ammonium sulphate reagent on TLC as shown in Figure 2d. The purity of the fungal vinblastine and vincristine showed similar chromatographic properties as that of the standard vinblastine and vincristine in three different solvent systems (a, b and c) on TLC.

### Purification and quantification of vinblastine and vincristine by HPLC

**Figure 3:**
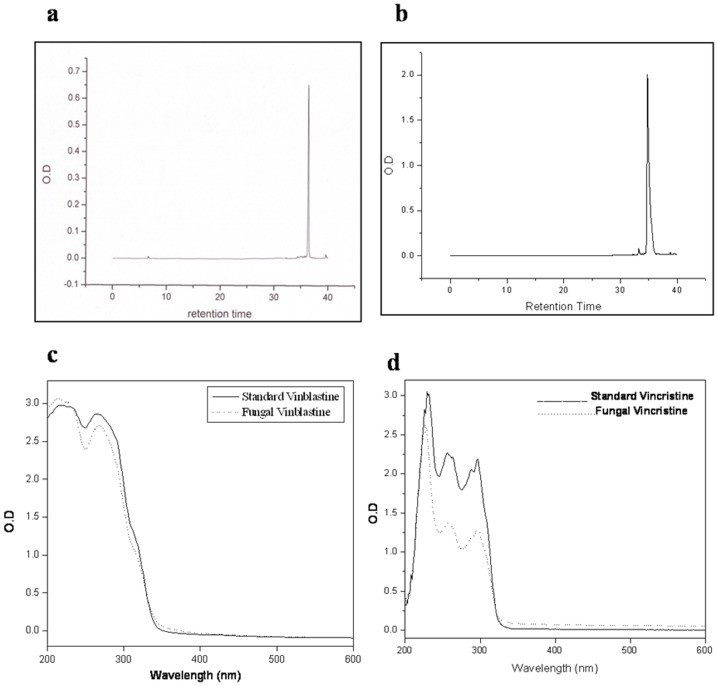
HPLC and UV spectrum. (**a**) HPLC profile of pure fungal vinblastine with retention time of 36.6 min. (**b**) HPLC profile of pure fungal vincristine with retention time of 34.9 min. (**c**) UV absorption spectrum of standard vinblastine and fungal vinblastine. (**d**) UV absorption spectrum of standard vincristine and fungal vincristine.

The homogeneity of the purified compounds was confirmed by HPLC analysis, which showed a single, symmetrical peak with RT 36.6 min (Figure 3a) and 34.9 min (Figure 3b) on C18 symmetry column of vinblastine and vincristine respectively [22]. Absorbance of the eluting compound showed high intensity at 220 nm and relatively low at 254 nm. The UV absorption analysis showed a peak representing absorption at 220 nm. The absorbance maximum of standard vinblastine and vincristine was also obtained for comparison and is shown in Figure 3c and 3d respectively [23]. The data of area peak vs. vinblastine and vincristine concentration, obtained in case of the standard sample was used to estimate the quantity of fungal vinblastine and vincristine. The isolation of these vinca alkaloids from 1 liter culture filtrate yielded 76 µg and 67 µg of vinblastine and vincristine respectively [24].

### Electrospray ionization mass spectrometry (ESI-MS) and Tandem mass spectrometry (MS-MS) analysis

**Figure 4:**
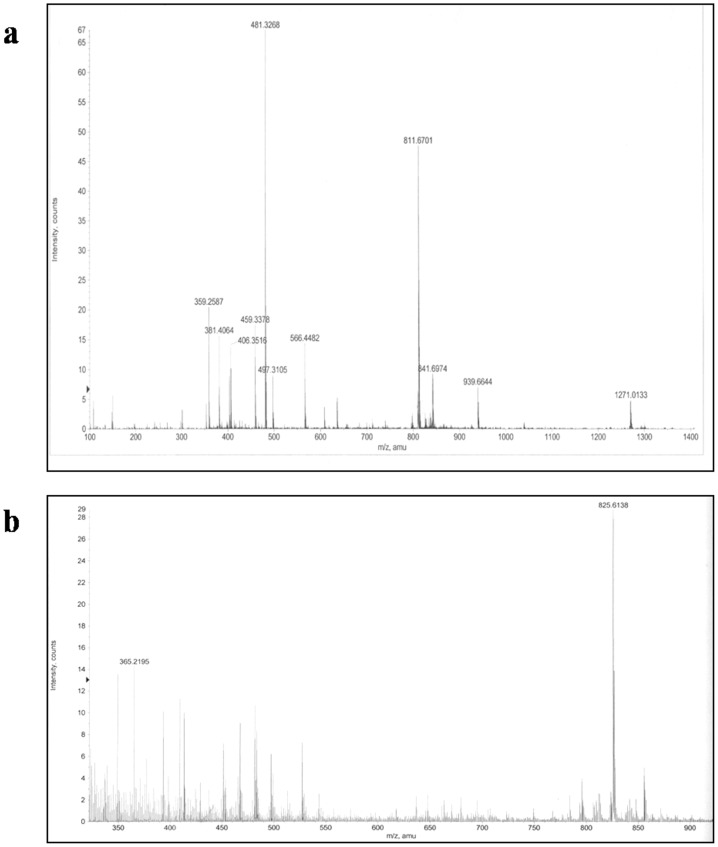
ESI-MS spectrum. (**a**) Molecular mass determination of the fungal vinblastine by ESI-MS. (**b**) Molecular mass determination of the fungal vincristine by ESI-MS.

**Figure 5:**
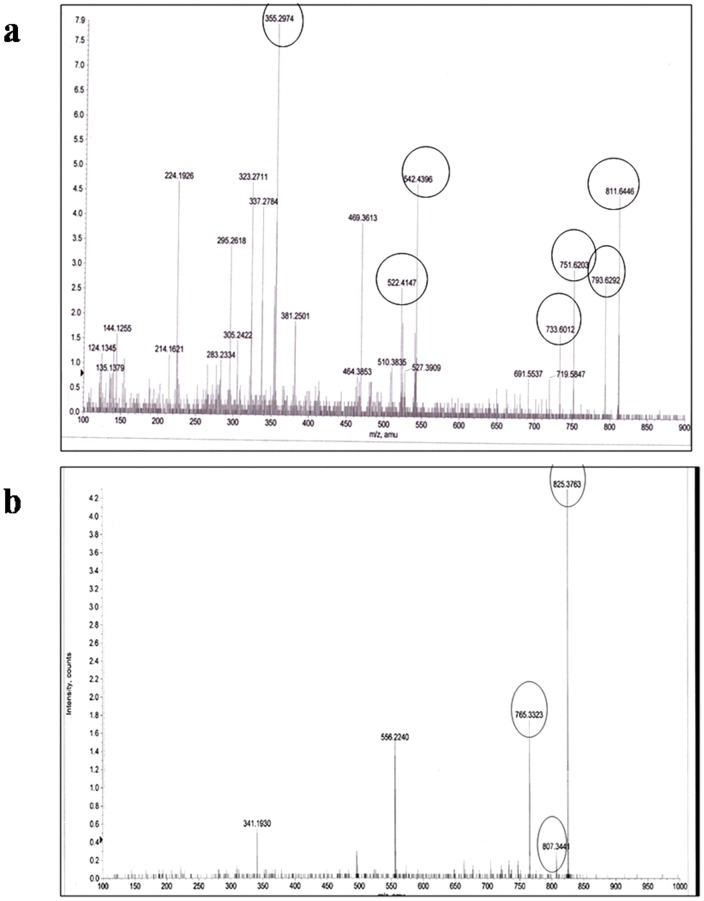
MS-MS spectrum. (a) MS-MS spectrum of the purified fungal vinblastine. (b) MS-MS spectrum of the purified fungal vincristine.

Electrospray ionization mass spectrometry yielded a major ion at 811 (Figure 4a). MS-MS spectrum of the purified fungal vinblastine showed product ions 355, 522, 542, 733, 751, 793 and 811 which were attributed to the (M+H) ion with m/z 811 (Figure 5a). Similarly, the electrospray ionization mass spectrometry of the purified fungal vincristine yielded at major ion at m/z 825(Figure 4b). In MS-MS, fragment ion at m/z 766, 807 and 825 were seen (Figure 5b) [25]–[26].

### ^1^H NMR analysis

**Figure 6:**
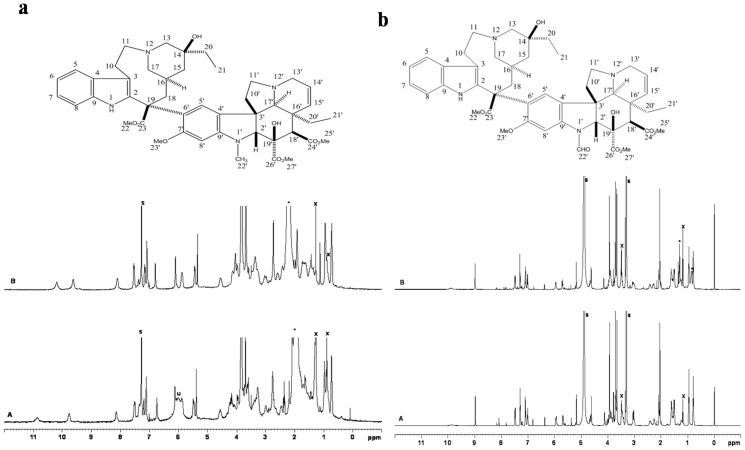
^1^H NMR spectrum. (a) 400 MHz ^1^H NMR spectra of fungal vinblastine (A) and standard vinblastine sulphate (B). The signals marked with ‘s’ is coming from the residual solvent (CHCl_3_). The signals marked with * and x are due to water (from the solvent) and contamination form n- hexane, respectively. The broad signal marked ú′ at ∼6 ppm in A is an unidentified impurity. (b) 500 MHz ^1^H NMR spectra of fungal vincristine (A) and standard vincristine sulphate (B). The signals marked with ‘s’ are coming from the residual solvent (Methanol-d_4_). The signals marked with * and x are due to contamination from trace amounts of n-hexane and ethanol, respectively.

A comparison of the ^1^H NMR spectrum of fungal vinblastine and vincristine and the standard vinblastine sulphate and vincristine sulphate (procured from Sigma Aldrich) are shown in Figures 6a and 6b respectively. The spectrum obtained for the fungal vinblastine and vincristine matches with the standard sample. The chemical shifts are summarized in Table 1 and Table 2 respectively. These values match with the chemical shifts reported in literature [27]–[30]. The minor changes in the chemical shifts are likely to be due to the fact that the standard samples are the sulphate salts of vinblastine and vincristine while the fungal vinblastine and vincristine are extracted as the base.

**Table 1:** Proton NMR (^1^H NMR) chemical shifts of standard vinblastine and fungal vinblastine in CDCl_3_.

Sr. No.	Vinblastine Sulphate Chemical Shift δ (in ppm)	Sr. No.	Fungal Vinblastine Chemical Shift δ (in ppm)
1	10.19	1	10.81
5	7.53	5	7.52
6	7.07	6	7.10
7	7.17	7	7.19
8	7.09	8	7.12
10a,b	3.03,3.38	10a,b	3.36,2.99
11a,b	3.38,2.97	11a,b	3.38,2.99
13a,b	2.74,4.57	13a,b	2.76,4.57
15a,b	1.44,1.51	15a,b	1.44,1.55
16	0.86	16	0.86
17a,b	3.4,2.4	17a,b	3.64,2.67
18a,b	4.15,2.42	18a,b	4.21,2.48
20a,b	1.30	20a,b	1.30
21	0.98	21	0.98
22	3.70	22	3.69
2′	3.98	2′	3.98
5′	6.80	5′	6.76
8′	6.10	8′	6.12
10′a,b	1.8,2.1	10′a,b	1.82,2.19
11′a,b	3.1,3.3	11′a,b	3.28,3.38
13′a,b	4.04,3.38	13′a,b	4.1,3.38
14′	5.89	14′	5.89
15′	5.44	15′	5.49
17′	2.70	17′	2.71
18′	5.36	18′	5.39
19′	9.36	19′	9.78
20′a,b	1.4,1.8	20′a,b	1.4,1.8
21′	0.73	21′	0.73
22′	2.73	22′	2.76
23′	3.81	23′	3.84
25′	2.10	25′	2.08
27′	3.84	27′	3.84

**Table 2:** Proton NMR (^1^HNMR) chemical shifts of standard vincristine sulphate and fungal vincristine in Methanol-d_4_.

Sr. No.	Vincristine Sulphate Chemical Shift δ (in ppm)	Sr. No.	Fungal Vincristine Chemical Shift δ (in ppm)
5	7.48	5	7.49
6	7.01	6	7.01
7	7.07	7	7.08
8	7.09	8	7.09
10a,b	3.47,3.69	10a,b	3.48,3.69
11a,b	3.32,3.47	11a,b	3.32,3.47
13a,b	3.03	13a,b	3.03
15a,b	1.23,1.17	15a,b	1.17,1.23
16	0.88	16	0.86
17a,b	2.40,3.83	17a,b	2.40,3.83
18a,b	2.28,4.65	18a,b	2.28,4.65
20a,b	1.50,1.62	20a,b	1.50,1.62
21	0.95	21	0.96
22	3.68	22	3.48
2′	4.62	2′	4.62
5′	7.08	5′	7.09
8′	7.30	8′	7.31
10′a,b	1.61,2.07	10′a,b	1.61,2.07
11′a,b	3.0,2.42	11′a,b	3.0,2.42
13′a,b	3.47,3.88	13′a,b	3.47,3.88
14′	5.94	14′	5.95
15′	5.67	15′	5.69
17′	3.34	17′	3.34
18′	5.18	18′	5.18
20′a,b	1.60,1.58	20′a,b	1.60,1.58
21′	0.79	21′	0.79
22′	8.98	22′	8.99
23′	3.93	23′	3.93
25′	2.03	25′	2.03
27′	3.65	27′	3.66

## Discussion

To the best of our knowledge, this is the first report of isolation of vinblastine and vincristine from an endophytic fungus *Fusarium oxysporum* isolated from Indian *Catharanthus roseus* plant. As earlier reported from other sources, the necessary precursors such as tryptophan and geraniol which are essential for the formation of vinblastine and vincristine were provided in the growth medium and 76 µg of vinblastine and 67 µg of vincristine is obtained per liter of culture filtrate. Thus, the total amount of vinca alkaloids (vinblastine and vincristine) using our fungal protocol is 0.0143% which is 2.86% more than the conventional plant-based method which yields 0.005% vinca alkaloids [31]. In order to increase the concentration of these drug from micrograms to grams per liter, efforts should be made to optimize the culture conditions by figuring out the biosynthetic pathway of the fungus *Fusarium oxysporum* along with genetic engineering methods which can lead to abundant production of these life-saving drugs. This green synthesis method is eco-friendly, non-toxic and cheap; unlike the chemical synthesis routes which were being used earlier and provided insufficient quantities of drugs.

In conclusion, we for the first time, have isolated an endophytic fungus *Fusarium oxysporum* from the Indian *Catharanthus roseus* plant which produces the anti-cancer drugs vinblastine and vincristine in 76 µg/lit and 67 µg/lit concentrations respectively, with properties similar to that of the plant derived vinblastine and vincristine. Since the fungus is producing vinblastine and vincristine, there are significant chances that this fungus may also produce other vinca alkaloids such as catharanthine, vindoline, secologonin, ajmalacine, etc. and may also be used for the fungal-mediated transformation of vinblastine and vincristine to other vinca alkaloids at room temperature itself. Also, there is a possibility that the fungus may synthesize some compounds with better anti-cancer properties than the present drugs. This method of production of drugs is in complete synchronization with the environment and when optimized, will lead to surplus production of these drugs, thus reducing their price and making them readily available.
